# Emerging Methods and Challenges Associated With Teaching and Learning Media Studies During the COVID-19 Pandemic Induced Lockdowns in Zimbabwe and South Africa

**DOI:** 10.1177/21582440231167113

**Published:** 2023-04-21

**Authors:** Albert Chibuwe, Allen Munoriyarwa

**Affiliations:** 1 University of the Free State, Bloemfontein, South Africa; 2 University of Johannesburg, South Africa; 3 University of Botswana, Gaborone, Botswana

**Keywords:** media studies, teaching technologies, COVID-19, South Africa, Zimbabwe

## Abstract

COVID-19’s arrival in Zimbabwe and South Africa in early 2020 caused disruptions to all facets of life including education. It disrupted traditional notions of media studies’ teaching and learning. In the contexts of these disruptions, the present study interrogates how selected universities in Zimbabwe and South Africa adjusted to the new normal in so far as teaching and learning of media studies is concerned. It is a comparative analysis of selected Zimbabwean and South African universities. In-depth interviews with students and lecturers and participant observations were used to gather data whilst thematic analysis was utilized to analyze the data. The study found out South African universities adjusted far much better and easily than their Zimbabwean counterparts. This is because both lecturers and students were capacitated as opposed to the scenario in Zimbabwe where lecturers and students alike were not given gadgets to smoothen the transition to online learning. The data that was given to lecturers was too little whereas the data for e-learning was too exorbitant for the students. Furthermore, both lecturers and students noted that it is difficult to teach and learn practical modules online. However, universities in both countries utilized platforms such as *Google* classroom though students from rural areas in both countries were affected by the digital divide.

## Introduction

The COVID-19 pandemic which led to national lockdowns, social distancing, and other related precautionary practices, was one of the most disruptive forces of this century. Universities across the world closed, giving way to long term adverse effects on education (Noor et al., 2020). They had to find ways of ensuring continuity in teaching, learning, and research. This was not an easy task for institutions that were used to face-to-face teaching and learning. The consequences of these disruptions were not similar across all universities (UNESCO, 2020). Some universities adjusted fairly and relatively easily (Cuschieri & Calleja Agius, 2020). This is because they had, over time, developed the technological infrastructure on which they could fall back on, as teaching and learning changed (Le Grange, 2020). Other universities, on the other hand, struggled to find ways through which teaching and learning could continue in the face of COVID-19.

This current study explores the emerging methods and challenges associated with the teaching and learning of media studies. By media studies, we adopt a liberal definition of the word, which encompasses all forms of courses ranging from communication, strategic communication, journalism, and film studies. Though the article has a special bias toward practical modules such as broadcasting, documentary film production, film and video production, and news writing etc., during the pandemic in South Africa and Zimbabwe, it also focuses on theoretical modules as well. It also seeks to understand the strategies universities put in place to deal with the challenges. The research seeks to answer the questions: What methods did selected universities in Zimbabwe and South Africa adopt in teaching media studies courses during the COVID-19 induced lockdowns? What challenges did both lecturers and students encounter in teaching and learning media studies during the COVID-19 induced lockdowns? The intention of this is to establish the ways in which media studies teaching and learning was impacted on by COVID-19. In attempting to answer the two questions we argue that in both countries the COVID-19 induced transition to new modes of teaching and learning media studies can only be understood through an extrapolation of historical factors—for example, the existence of supportive digital infrastructures in universities that could easily be expanded. We also argue that the political and economic conditions obtaining in a country determined how universities, lecturers and students adjusted. Lastly, our data help us sustain the argument that the relationship between the students, university management, and staff played a crucial role in either mitigating or aggravating the challenges associated with teaching and learning media studies courses. Our paper contributes to burgeoning literature on media studies pedagogy in the global south (see Banda et al., 2007; Mano, 2009; Tomaselli et al., 2013). However, Scopus Index, *Google* Scholar, and ProQuest searches show that there is no scholarship that explores how teaching and learning was altered to accommodate the burden the pandemic placed on media studies teaching and learning in South Africa and Zimbabwe.

## COVID-19 and Media Studies Education

Research at the intersection of media studies education and the COVID-19 pandemic is still scarce. In Africa, there is a small intellectual corpus on media studies education and the quality of the graduate (Motsaathebe, 2011). In Zimbabwe, existing research argues that (media studies) education needs transformation, as it is not fit for purpose (Information and Media Panel Inquiry [IMPI], 2014; Matereke, 2012).

At the center of existing studies on media education in both Zimbabwe and South Africa is “the old question relating to what is perceived as the best way to prepare journalism students” (Motsaathebe, 2011, pp. 385–386). But given the advent of the COVID-19 pandemic we, perhaps, need to reframe the question(s). This is because COVID-19, like any other crisis, unsettled norms (see Perreault & Perreault, 2021) including in the education sector. In the USA, just like in Zimbabwe and South Africa, “The…COVID-19 outbreak resulted in the closing[,]…in March of 2020, [of] most major universities and colleges…students…were informed within two weeks the remaining of their Spring 2020 semester would be moved to… online format…” (Unger & Meiran, 2020, pp. 256–257). In neighboring Botswana (see Ntshwarang et al., 2021), the University of Botswana went online but there were issues of poor or lack of internet access off campus, reluctance by employees etc. In South Africa and Zimbabwe, universities as well as almost all businesses closed on 27 and 30 March 2020 respectively, as the countries went into the first lockdown. This disruption caused by COVID-19 to university education in particular and education in general means that, “…changing the way we teach our students to prepare for a “new normal” may be necessary,” (Sweeney, 2020, p. 34). In other settings teachers and lecturers quickly took crash courses in online teaching (see Sweeney, 2020). As Sweeney (2020) notes,

Many questions still linger about the future of the virus and its impact on student learning, and the lasting impact and changes in the journalism industry… Making sure J-school pedagogy quickly adapts to the “new normal” will be paramount in preparing our students with the needed skills to adapt to whatever the landscape becomes on the other side of the pandemic (p. 36).

The “many questions” have resulted in a burgeoning scholarship that seeks to understand the future of COVID-19 and “its impact on student learning” (Sweeney, 2020, p. 36; see also Dutta, 2020; Hodges et al., 2020; Ntshwarang et al., 2021; Unger & Meiran, 2020; Wotto, 2020). In the light of the above, journalism school pedagogy has to, arguably, adapt quickly to the new normal of online teaching and learning. However, how feasible is this in a context of political and economic problems (Zimbabwe) and economic inequalities (South Africa)? Furthermore, how feasible is it to hold effective online lectures with huge classes (see IMPI Report, 2014)? What challenges did universities, lecturers, and students face in teaching and learning media studies courses online? What measures did the students and lecturers alike put in place to navigate through the challenges? But with South Africa the political-economic and technological situation is different. Their economy performs way better than Zimbabwe’s whilst their politics is not as volatile as Zimbabwe’s. Given these contextual differences, what are the likely differences and similarities in the teaching and learning methods deployed, the challenges faced and the strategies used to overcome them?

## Theoretical Framework

This paper is grounded in tenets of the disruption theory (Christensen, 1997). A disruption represents a sudden and often unexpected interruption to a status quo (Amada, 1994). Following on this, we argue that COVID-19 and its attendant prevention protocols such as social distancing, sanitizing, lockdowns, wearing of masks etc. was a disruption that upset the norm in higher education and every facet of life. Face to face lectures were rendered impossible and highly dangerous by the pandemic. However, just like all disruptions that often always precipitate change to current systems in higher education (Robinson, 2017), COVID-19 was also a precipitated the digital turn in higher education. Higher education systems are often static and anachronistic (Robinson, 2017) but COVID-19 forced them to be dynamic. It is arguable that higher education, infamous for failing on many occasions, to respond adequately to disruptions or changes obtaining in the economic, socio-cultural, political, and digital spheres (Amada 1994; Denning, 2016; Robinson, 2017); had to either innovate or perish from the COVID-19 induced disruptions. It is there important to interrogate how higher education, specifically in the area of media studies teaching and learning, adapted during COVID-19 given its historical failure to respond to disruptions.

In the light of the foregoing, there are key tenets of the disruption theory that we utilize in this paper. Firstly, disruption is always a consequence of the battle between incumbents and challengers in a particular field (Denning, 2016). From this perspective, we understand the COVID-19 pandemic as one such disruption that challenges traditional ways of media, journalism, and communication teaching and learning. Disruptions may cause threats to a field (Christensen, 1997). If the disruption is massive, it might cause seismic shifts in certain field practices (Denning, 2016). However, organizations make strenuous attempts to keep their clients in their confidence and offer solutions (Christensen, 2017). This means, they respond to the disruption and still continue to offer a service. This is important in this paper as we seek to examine how universities responded to the COVID-19 disruption while trying to continue offering education to their students. Secondly, disruptions may trigger a reaction from targets of the disruption who may innovate and provide an even better service (Amada, 1994). But this entirely depends on the level of disruption as well as exogenous factors like the political, economic, relational, and social factors obtaining in the context where the disruption happened. Disruptions can either be internally or externally induced, however, COVID-19 was an externally induced disruption. In some instances, disruptions may be so massive that targets may have no way of coping, and may end up being destroyed at worst, or struggle to acclimatize (Amada, 1994). Christensen (2017) sums it thus, “Disruption is a process, not an event….” (p. 34). The same is true of the COVID-19 disruption. In this context, we focus on the challenges (or disruption) presented to universities, lecturers, and students in teaching and learning media studies modules by COVID-19 and the methods and, or strategies the universities, lecturers, and students adopted to deal with the disruption or challenges brought by COVID-19.

## A Note on Methods

In this paper we integrated several qualitative approaches that synced with the ravaging pandemic and our own circumstances. We utilized participant observation, interviews, and informal conversations with colleagues and in the Zimbabwean case, with students as well. Participant observation and informal interviews were utilized in the South African case, while interviews and participant observation worked in the Zimbabwean case. Participant observation enabled the researchers, both media studies lecturers, to learn about media studies teaching from their own environment. The researchers work in the South African and Zimbabwean university contexts. The advantage of such an approach to us was that we had a better understanding of the context and the phenomenon under study. Participant observation increases validity of the study and it becomes even stronger when integrated with other approaches (DeWalt & DeWalt, 1998). In the South African case, we augment participant observation with informal conversations; what Bernard (2011) calls, conversation with a purpose. Informal conversations are about talking to people within a certain context to gain insights into a practice and generate valuable data (Swain & Spire, 2020). It offers an opportunity to expand and enrich data by engaging in both structured and unstructured conversations. Further, informal conversations offer context and authenticity to data (Swain & Spire, 2020). We, however, need to note that informal conversations have their ethical issues (Atkinson et al., 2018). For example, in this particular research, should participants be informed? This research is however, not sensitive. Secondly, the dignity and privacy of the people who we engaged with in informal conversations remain unaffected. The institutions they work for were neither disparaged nor exploited. More importantly, our informal conversations did not come to any physical, psychological, and emotional harm—which are the most important ethical principles in research (Delamont & Atkinson, 2018). The informal conversations allowed the researcher to observe and gain insights from participants in their natural settings. More importantly, it is not obtrusive as a method, yet, simultaneously, it allows researchers to make pertinent observations that can adequately inform a research (Swain & Spire 2020). In Zimbabwe, online interviews were used since we had been dispersed by the COVID-19 induced lockdown. The interviews were conducted with three colleagues, two lecturers, and one technician, in the Department of Media and Society Studies at the Midlands State University. Ten students in the second and fourth years were interviewed. We did not interview first year and third year students because at the commencement of the lockdown we did not have any level one students whilst the level three students were away on industrial attachment. The Zimbabwean interviews were conducted between 01 and 24 December 2020. Thematic analysis was utilized to analyze data but instead of using themes emerging from literature review we relied on themes emerging from the data to arrange and interpret it (see David & Baden, 2017; Fereday & Muir-Cochrane, 2006). We use, in the analysis and or interpretation, verbatim quotes from respondents to ensure that the interpretation “remains directly linked to the words of the participants” (Fereday & Muir-Cochrane, 2006, p. 3).

## Insights From South Africa’ Higher Education

Broadly, the universities we observed and the lecturers we had informal conversations with adopted three approaches in teaching various practical communication, media and journalism courses during the COVID-19 induced lockdowns. These three approaches are: (a) integrating media technologies in the media curriculum, (b) reinforcing specific practices and pedagogical behaviors, and (c) shifting and building resilience.

## Integrating New Media Technologies

The findings show that South Africa’s leading universities (University of Cape Town, University of Witwatersrand etc., according to the Times Higher education, 2020), easily adapted to the COVID-19 disruptions. We observed, for example, that the University of Johannesburg. which is leading contemporary debates on the Fourth Industrial Revolution (4IR), made a quick and comfortable switch to online learning. From our observation, the reason is that this university was already a step ahead in terms of embracing technology in pedagogy. It, therefore, had a “smooth landing” when the disruptions came. Through, for example, the Blackboard learning facility at… (name of university removed to enable blind peer review), journalism students continued lectures with minimum-to-no interruptions. It was not difficult for students to gel into these newer practices quite easily because prior to the outbreak, they had utilized these platforms, albeit with less frequency. The same can be said of the University of South Africa (UNISA); one of the biggest online learning universities in Africa (Mashile & Matone, 2012). And, like… (name of university removed to enable blind peer review), the communication department at the university drew on the historical capital of its well-established online presence to retain a pedagogical normalcy. It was easy for South Africa’s universities to adopt new technology mediated teaching methods because they are relatively well-resourced compared to universities in other parts of Africa and even most in the global north (Cloete & Moja 2005). They thus borrowed from what Christensen (2017) calls “historical capital,” that is, their strength and the resources they already have, to counter the massive disruption caused by COVID-19 and retain normalcy in their operations.

COVID-19, arguably, led to a fully-fledged and more focused use of online platforms in media education. As one lecturer noted, media departments have always been at the forefront of online teaching and this made their switch to online platforms easy. What we observe in the South African context is that an integration of technology in teaching media led to an equal push for network-mediated collaborative learning. One lecturer argued that this shift to technology-mediated learning represented an equally revolutionary paradigm shift on the part of the students of journalism where they had to understand that online teaching was no longer a question of choice, but imperative practice that had no substitute. On the part of the lecturers, online learning enabled them to experiment with new ways of teaching and learning both theoretical and practical modules. As noted by one lecturer, the new mode of learning has enabled lecturers to coordinate media teaching and learning material in newer ways that speak to both media theory and practice. These are ways or methods they had not done before the pandemic. For instance, lecturers point to courses like documentary filmmaking, which one lecturer said could easily be taught in both its theoretical and practical aspect as one. He said, “students could easily upload short documentaries on the online system where they, simultaneously were able to download class notes, slides and related readings.” As Lecturers pointed out, the success of this online practice lies in many university lecturers and students embracing and reinforcing certain practices and behaviors. However, the lecturers also noted that students had difficulties adjusting to the new mode of online learning. This could be because it does not matter how well-prepared organizations are as there will always be evidence of shock when they encounter disruptions (Denning, 2016). In this case, the shock lies in students having to learn new ways of participation.

## Embracing and Reinforcing New Teaching and Learning Methods

In this section, we note that for a successful transition to online teaching and learning, both the staff and students of media studies had to reinforce new pedagogical practices and behaviors.

The first method that was reinforced in teaching media is asynchronous learning. By asynchronous learning, we mean a scenario where lecturer and student are not in class at the same time; teaching and learning happens at different times. But the students will get the pre-recorded lecture together with study materials (Chaturvedi et al., 2021). As one journalism lecturer remarked, “We had to meet the student on the platforms where they are available.” What we observe is that lecturers responded to students via emails, and social network platforms. They also used several experiential learning tools like *YouTube* videos and blogs. The overall effect of such an approach was that it made lecturers adopt a learner-centered approach in unprecedented ways. However, the challenge for the lecturers lies in how to deliver practical courses online. One respondent, a journalism lecturer stated, practical modules were delivered via video conferencing platforms embedded on online teaching and learning platforms.

The second method that was reinforced in teaching media-related courses is collaborative learning. Collaborative learning was a function that had been embedded in digital teaching platforms. But, it had rarely been exploited as lecturers preferred physical presence of the teaching and learning. We observe that the group function of the Blackboard teaching and learning platform at [name of university removed to enable blind peer review], became a popular instrumental feature. One of its advantages is that it allows lecturers to utilize pre-existing groups, or form breakaway groups quite flexibly even during the class. In addition to this type of collaboration, we observed that the pandemic increased the collaboration between lecturers and their students’ parents and guardians. This relationship has in previous times, at least in South African universities, never risen beyond “chance encounters.” But, as we note, the pandemic heightened parents’ and guardians’ interest in their children’s learning. One lecturer noted that at one point, he had to engage with the guardians of some of his students who were inquiring about the performance of their children and their grades, especially in the practical modules. As he explained it, “it was awkward as…marks are confidential, and we have agreements with the student, not the guardian or parent. But you could not help marvel at the concern that a parent had shown….” This was a positive form of collaboration that contrasted with the pre-pandemic “aloofness” of South African parents and guardians when it comes to their children’s learning at universities (see Nel et al., 2014). Before COVID-19 they had always seen themselves only as providers of the educational material needs of the child (Nel et al., 2014). But due to the pandemic parents and guardians were “brought back into the circle” of their children’s’ learning through helping them adjust to the new learning environment. One lecturer asserted that, “Parents could not avoid this process as much as they wanted to…remember at the height of lockdowns some parents were also home. It was no longer a chance and accidental encounter…” This had a huge effect on the learning behaviour of students as we explore in the section below.

## Shifting Attitudes and Building Resilience

For a start, parents and guardians had to help their children adapt to the online learning environment. And parents and guardians had to reconcile to their children’s ever-presence at home when most were used to them being at school. This, obviously exerted a significant amount of mental pressure and terror on both parents and students. On their part, lecturers had to do the extra work of building resilience amongst students. Here we take resilience to mean, “…having the ability to have a successful outcome despite being in a challenging situation” (Masten et al., 1990 as cited by Chaturvedi et al., 2021, p. 5). For South African lecturers, this was made easy by the involvement of parents. One lecturer asserted that, “journalism could be overwhelming. It has practical components that had to be unpacked in class first. But because this could not happen, students felt the loss of the physical class where they could ask questions before embarking on the practical itself…” Lecturers had to work toward ensuring greater involvement of students, to motivate them so that students can ignore the ravaging pandemic and its overwhelming disruptions and focus on the task at hand.

The students also had to be there for each other; they had to constantly cheer each other up especially with the challenging practical courses. However, we observed that verbal fights among students were common. For example, for a film studies group, a common cause of verbal fallouts was how to organize practical work: who will pick up the film equipment from the university film labs, considering that due to shelter-in-place regulations, the whole group could not go? Who will shoot which part of the short film? And who will return the equipment? The first and last questions were even more emotive considering that they involved personal transport and time expense and personal risk. No student was ready for such risks, and this often created tension among students.

For lecturers, this often came with two responses—a heightened sense of duty to care and, what we call here “embedded media teaching approaches.” The duty to care involved many activities on the part of the lecturers. They had to, for instance; engage in dispute resolution; explain and clarify issues more frequently than ever. They had to constantly motivate faltering students by “cheering them up” as one lecturer noted. This was an extra responsibility, albeit one which was inevitable during the pandemic. The duty to care precipitated a new teaching approach; “embedded teaching.” Lecturers, from what we observed, joined students’*WhatsApp* groups. This was meant to be “closer to the student, and the action.” We noted that *WhatsApp* assumed a greater responsibility as a platform where instructions could easily be passed, and where the lecturer could engage with students. Its ease of access and flexibility of use helped lecturers. But this also represented two major developments. First, it represented the increasing “mainstream role” that *WhatsApp* assumed as a platform for pedagogical instruction—a role it never had before the pandemic. Thus, it has become a formal space, integrated in the teaching and learning processes. Secondly, it however, represented an inevitable invasion of the “class/group privacy.”*WhatsApp* groups arguably always existed for students. The *WhatsApp* group was a platform “fenced off” from lecturers, and only used by students to communicate learning issues, and to vent their frustration against the lecturer, the system, and everyone else, in the absence of authority. Students always used the spaces to speak back to power because it was their space. One student cynically remarked, “When lecturers join our *WhatsApp* groups, they make it difficult for us to express ourselves, we feel their authority even on those groups. That is why we would form another class group which excludes them…” Ironically, it also added a responsibility on the lecturers to provide constant feedback and direction using those platforms.

## The Zimbabwean Experience

This section presents findings from Zimbabwe. It is divided into three sections namely: Use of online platforms and how they were deployed; challenges and or disadvantages of online learning in an economically depressed environment and; advantages of online learning.

## Old Habits Die Hard: Hybrid Approach to Media Studies Teaching and Learning

The findings showed that in Zimbabwe, at…University [name removed to allow blind peer review], a blended approach was used. Both traditional face-to-face and technology-based methods were utilized. Both students and lecturers stated that *Google* classroom, and *WhatsApp* were mostly used for teaching and learning during the COVID-19 induced lockdowns. Module outlines, notes, reading materials, and assignment questions were circulated via *Google* classroom and *WhatsApp*. However, lecturers noted that the institution’s preferred platform was *Google* classroom but they had to use *WhatsApp* to cater for those students with no access to *Google* classroom due to lack of data or smart phones/laptops. Lecturers stated that most students failed to attend the *Google* Classroom lectures as a result they pleaded with lecturers to also use *WhatsApp*. As Lecturer A noted, “Generally, students did not have data to access *Google* Classroom, so I had to pivot to *WhatsApp*, where I have options of typing and voice notes.” The attendance of students in *Google* class lectures was much less than in physical classes. For example, in one class of about 100 students, *Google* classroom attendance averaged 15 to 20 whilst in another of about 250 the attendance averaged between 25 to 30 students per lecture. The reasons for this massive absenteeism shall be explored in the next section. The *WhatsApp* lectures took the form of live discussions that combined use of voice notes, live texting, sharing of short video and audio presentations, and reading materials and lecture notes. For example, one of the researchers made use of 10 to 15 minute pre-recorded audio-visual, and audio seminar presentations with his Master’s classes. The students used their smart phones to record and compress the presentations. The lectures utilized the seminar method in which these videos and audios were uploaded and viewed or listened to during the live *WhatsApp* lecture. The viewing and or listening to the presentation would be followed by a session in which fellow students would ask questions and make contributions. The lecturer’s intervention followed and this was concluded by giving students a chance to ask questions or make contributions. This involved a lot of typing, and voice notes. The *WhatsApp* lectures complimented *Google* Class lectures and submission of written presentations and assignments via email or *Google* classroom platform. This was mainly feasible with theoretical modules as the lecturers interviewed argued that teaching practical lessons online was difficult. However, Technician A who helps with practical film and video production, and television and radio production demonstrations stated that, “creating and uploading videos [on *WhatsApp* and *Google* classroom] with me demonstrating…plus voice notes and prepared text notes” was the solution. But in a context where students had no access to cameras, computers and the necessary software for editing one wonders whether this worked.

For the lecturers who found it difficult to teach practical modules online the alternative was to wait for the opportunity to teach the students in face-to-face lectures. The university implemented a phased approach in which students came back to campus for exams in batches. Their time on campus ranged from 2 to 4 weeks and was inclusive of a week for revision for the classes with theoretical modules and 2 or 3 weeks for the classes with practical modules. The extra week or two for classes with practical modules was to give them ample time to produce their projects. Lecturer A stated that, ““But on practical issues such as lighting, camera (cinematography), editing, directing and acting, online [teaching] is quite useless. So for practical work, I had to wait for students to come to campus to film and edit under my supervision…Also students have to use similar equipment so that marking is fair, otherwise those not affluent and [who] don’t own cameras or top-of-the-range phones would be at a disadvantage.” Lecturer B stated that, he “did some of the introductory parts using online platforms and held group sessions when students returned to campus for revision and exams.” It is apparent that for the two lecturers practical modules such as film and video production, and radio and television production could not be taught online due to the need to teach the students how to operate gadgets such as the camera. The foregoing findings demonstrate that a blended approach which utilized both online (*Google* classroom, *WhatsApp*, e-learning, and email) and offline teaching and learning methods was used. The findings also demonstrate that lecturers adapted to the situation depending on their own individual assessment of their students’ needs. Consequently, even though the university preferred *Google* classroom and e-learning platforms, lecturers’ appreciation of the majority of students’ lack of smart phones, laptops, and money to buy data bundles for the *Google* class/online lectures led them to also utilize the much cheaper *WhatsApp* platform. *WhatsApp* ceased to be merely a platform for socializing and became a tool for teaching and learning where its features such as instant messaging and voice notes were utilized for live presentations and discussions by both lecturers and students.

## Methodological, Psychological, and Contextual Challenges of Online Learning in the Zimbabwean Context

The findings show that there were quite a number of pedagogical, psychological, and contextual challenges associated with online learning. The findings reveal that whereas lecturers noted that the institution’s preferred platform was *Google* classroom, they had to compliment it with *WhatsApp* to cater for students with no access to *Google* classroom. For example, lecturer A stated that, “Generally, students did not have data to access *Google* Classroom, so I had to pivot to *WhatsApp…*” Access to *Google* classroom was also limited by connectivity and sometimes gadget-related issues. In other words, some areas have poor internet connectivity whilst some students either had no data or smart phones or computers to enable them to go online. For those with smart phones but had no money for internet data bundles, *WhatsApp* was the most conducive platform. The students pleaded with their lecturers to also use *WhatsApp* to cater for those with no money for data bundles. The students observed that data was expensive and unaffordable for most of them. The issue of poor internet connectivity and expensive data bundles were the most cited challenges associated with online learning. The students also pointed out that the home environment was not conducive for online learning. The reasons for this were cited as electricity outages, and household chores. Student D, a level 2 female student, noted, “network connection was a problem. Secondly buying data bundles was expensive and last but not least the environment at home was not conducive for learning and studying.” Student C stated that, “Home is never a school environment especially when you don’t even go out for classes. I personally missed most of the lectures because sometimes I would be doing the chores…. Electricity was also another challenge; sometimes the lectures would be conducted the same time when there is load shedding.” Student A, a level 4 student, noted that, “there were challenges in terms of data. Data is quite expensive and also being at home and balancing house chores and schoolwork was quite a challenge.” This was also echoed by Student B, who claimed that “there were network problems and some of us could not afford data for the discussions there was a serious network problem some messages would reach your phone only when the lecture was over and some of us didn’t even get the messages (sic).” However, most of the students who raised the challenges associated with household chores were female even though a few male students also raised the issue. For example, Student G stated, “most of the times I had no money to purchase data and also at times I could not attend online lectures because I will be doing gardening etc.” These disruptions associated with the online learning in the home environment reflect gender fault lines where the females were mostly preoccupied with chores in the home whilst the male students were tied up with chores outdoor. For example, ladies were mostly preoccupied with cooking, laundry, and cleaning whilst male students were tied up with gardening. The challenges are also reflective of the digital divide and the economic challenges in Zimbabwe where internet data bundles are very expensive and thus considered a luxury by families struggling to put food on the table.

Social media was also viewed as a potential distraction to online learning whilst psychological effects were also noted. For example, student C stated that, “There is a greater chance for students to be easily distracted by social media or other sites. Students can learn a lot from being in the company of other mates, however, in an online class, there are minimum physical interactions between students and teachers. This often results in [a] sense of isolation for the students.” For this student, social media itself is a distraction whilst the absence of interactions psychologically affects the students as they are denied the chance to physically interact with their classmates and socialize. It was also argued by the lecturers that, “Levels of participation and concentration are not measurable. One can just log on and leave the phone. No benefit of face-to-face [interaction] such as non-verbal communication, for example, frowning students show they are not understanding, and you explain in simpler terms. Online it’s not there.” Indeed, one undergraduate student confessed to one of the researchers in an informal conversation that, “during one online lecture in which you asked me to respond to a question, I was sleeping. I just logged on and went back to sleep.”

The failure to secure data was not only a challenge for the students as it also affected the lecturers. The failure by most students to attend online lectures meant that the lecturers had to create space and time for those that missed out on the *Google* class lectures. These were catered for via *WhatsApp* lectures and face-to-face lectures. The university, as noted earlier, provided a week and sometimes 2 or 3 weeks for face-to-face lectures. As one of the students noted, students who missed online lectures were catered for through face-to-face lectures just before examinations. This, arguably, demonstrates that online learning increases the workload for the lecturers. As one of the respondents, Technician A stated, “Data and gadgets determine the length and quality of [a] lecture, which had me default to *WhatsApp* instead of *Google* Classroom. Some students could be offline due to various constraints such as failing to possess smartphones or data.” This was corroborated by Lecturer B who also teaches practical modules who noted that, “Most students could not afford data. Some received messages well long afterwards because of network challenges hence could not ask questions where they needed clarification.” The problem of network connectivity also affected lecturers. One of the researchers experienced poor internet connectivity with the workplace issued mobile line but it improved somewhat after he contacted the service provider and asked them to upgrade the line to 4G. Lecturer B also stated that the, “main disadvantage [of online learning] is that both students and lecturers usually face data and network challenges. Also, [it is] difficult to monitor … concentration and participation from the students.” It was also difficult to monitor whether students are in class or not as some just log in and go away. Others also do not participate which raises the question of whether they will be present or not. It was also noted by lecturers that, fatigue and the “impracticality” of teaching practical modules online were the other challenges they encountered. As Lecturer A noted, online teaching is “Quite useless for practical modules such as film making.”*WhatsApp* is tiresome because it involves a lot of typing and given the huge classes, email, and *Google* Classroom are equally straining.

The requirement to conduct face-to-face crash lectures for those that missed out on online lectures not only exposed the lecturers to potential COVID-19 infections and possible death but also exposed the students to the same. Indeed, during these face-to-face lectures in mid-2021, colleagues and students alike were infected and affected by the COVID-19. It was also noted that the performance by the students was generally poor maybe because they are used to face-to-face lectures and not virtual lectures. Physical contact was minimal (1–2 weeks) and the students would be in lectures from 8 am to 5 pm. Those that taught them in the afternoon taught tired and hungry students. The challenges encountered by students in making the switch to online learning were compounded by the absence of assistance from government, the university, and the mobile network service providers. All the students noted that neither the government nor the university put in place measures to make smooth the transition to online learning. For example, Student A noted that, “There was…poor response [by the university] towards online learning compared to other universities that went as far as giving free data bouquets for students to access online learning platforms. Had it not been that our lecturers were understanding *(sic)* to have us make use [of]…easily accessible learning platforms…it could have been disastrous. There was also poor engagement between the university administration and students to determine the most effective way forward…I feel like they would (sic) have done better in communication and in engaging students to plan for a student concerned (sic) approach to accommodate all students.” Student F noted that, “The University did not put any measure in place to ensure full participation in online learning.” Lecturers were initially only given 19 GB which was reduced to about 9 GB and eventually nothing (as at the end of June 2021). The university did not provide gadgets for lecturers to enable online learning. The university however, held workshops with lecturers on how to use *Google* classroom for teaching. They also produced audio-visual tutorials which were shared via the staff email.

However, students and lecturers/technicians noted that, the above challenges notwithstanding online learning had the following advantages: students have simplified notes/study packs that they can easily refer to; online teaching and learning is flexible and it prevents direct contact during the COVID-19 pandemic thereby eliminating the possibility of infections.

## Discussion

This paper sought to examine the ways media studies teaching responded to the pandemic in different universities in Zimbabwe and South Africa. We have noted three main approaches in the South African case. These were: integrating new media technologies, and embracing and reinforcing new pedagogical practices and behaviors, and shifting attitudes and building resilience. In the Zimbabwean case, we noted the use of a blended approach that utilized a combination of *Google* classroom, face-to-face, and *WhatsApp* lectures—the latter was deployed by lecturers to cater for students with no access to *Google* classroom. On these platforms methods such as seminar presentations, audio-visual presentations and demonstrations, and live *WhatsApp* discussions combining voice notes, images, videos, and instant messaging, were used. The methods were inspired by a desire to cater for all students and they did not always reflect the institutional directives as lecturers adapted to the needs of the learners. What we noted was that in both countries, responses were dictated by economic, political, and social factors. In South Africa, we note that a relatively stable economy meant universities could utilize both their own resources and support from government and other stakeholders to ensure a smooth transition to online teaching. Secondly, we noted that a culture of consultations and inclusion in South African universities, among students, staff and university management, enabled them to transit smoothly to online teaching that thrived on historical infrastructures that lie dormant but not extinct in universities’ online platforms. However, the Zimbabwean case presented a culture of top-down or “commandist approaches” between management and staff and between management and students whilst between lecturers and students there was a culture of consultation. This could be because of the shortage of resources or it is simply reflective of the broader political and management cultures in post-2000 Zimbabwe that tend to gravitate toward the authoritarian.

We note that universities in South Africa drew on goodwill existing between them and other important stakeholders. For instance, some network service providers in the country provided zero-rated academic sites which students could access without data. This was a culmination of good relations that always existed between universities and network providers. This was unprecedented cooperation that, we argue, could not be cultivated overnight. It had to be based on pre-existing relations. But in the South African case, we notice that universities and Mobile Network Operators (MNOs) found it easy to work together in providing data because of government intervention. The South African government and MNOs had frequently quarrelled over the release and distribution of spectrum (Moyo & Munoriyarwa, 2021). But we note that when MNOs agreed on providing cheap data to students and making some platforms free, it was an expression that the three stakeholders could work together in such difficult times. In addition to this, we argue that a cordial working relationship between university management and teaching staff allowed for this smooth transition. South African universities have always maintained a cordial rapport with staff. It was easy for staff to respond to the call to move online. Thus, universities were drawing on historically good relations with their staff. In the case of South Africa, there are three issues we need to interrogate in this discussion.

First, we need to critique the cultural responsiveness of online teaching and learning methods. South Africa is one of the most unequal countries in the world (Moyo & Munoriyarwa, 2021; World Bank, 2018). Issues of inequalities and inequities still ring high and loud in the post-apartheid regime. This means some students, despite having 30 GB of data, could still be excluded form online learning for two reasons. Firstly, the pandemic “condemned” them to poorly connected rural areas. Secondly, considering that most are registered in a number of courses, 30 GB would not be enough and the poor and disadvantaged students’ families were in no position to augment university efforts.

Second, issues of evaluation online were prominent on journalism courses. How would online teaching be evaluated? In other words, what kind of a virtual evaluation methodology would work for online learning environments? Even though, as we noted, media courses in South African universities had embraced online learning and teaching before the pandemic, they had not yet, as we observed, come up with well-constructed virtual evaluation methodologies and tools (VLE). But, the fact that they had experimented with online teaching provided lecturers with a positive starting point. As one lecturer noted, “it was a question of repurposing the little we had prior to the pandemic to a larger group of students…”

Third, we interrogate is what we can loosely call the social processes of media pedagogy. In most media courses, students work on minor media projects. This required a new approach of managing students, support group practice work and interactive learning. But as we noted, universities quickly moved to provide instructors with the technological infrastructures that allowed them to manage huge classes online and retain control. For example, as we noted with UNISA and [name of university removed to enable blind peer review], students could engage in case study discussions online, role play online, fill information gaps online and many other activities.

The Zimbabwean case represents an entirely different scenario. There was no co-ordination between institutions of higher learning and learners. As the findings reveal, the students noted that they got nothing in terms of support from either the university or government. There was no coordinated effort between universities, government, and mobile network service providers to smoothen the transition to online learning. The only form of support the students got, they argued, was from lecturers agreeing to compliment *Google* Classroom lectures with *WhatsApp* lectures. The lecturers agreed to go beyond their mandate of teaching strictly via *Google* Classroom to also teach via the more affordable *WhatsApp* platform. This could be because the lecturers empathized with the students more since they too, other than data, a mobile line and tutorials on *Google* Classroom management, did not receive much by way of support to make them teach online effectively. Most, including one of the researchers, had out-dated laptops and smart phones that were not efficient for purposes of online learning. There were also sentiments that the data provided by the institution was inadequate whilst connectivity was not that very good. On their part, the university provided an opportunity for those that missed online classes to have crash face-to-face lectures just before examinations. Whereas this greatly helped the disadvantaged students, it presented two challenges namely; the possibility of COVID-19 infections and; overworking on the part of lecturers since they had to teach the same things online and offline. Interviewed lecturers and technician(s) expressed fears of infection during these face-to-face lectures. There was thus a general feeling of neglect by both university authorities and government amongst students and lecturers. It could however, be that both the government and universities were ill-equipped to respond to the COVID-19 induced disruptions given the well-known economic challenges that the country has been experiencing post-2000.

There is need to note a few issues about education digital technologies that we have examined in the case of South Africa and Zimbabwe. We have noted that the adoption of education digital technologies, especially in South African universities, provided a quick solution to the pandemic-driven disruptions. We noted that the right technologies, if deployed correctly int specific contexts, might provide a quick solution to problems of access. But, in both countries where social and income inequalities are real, the questions of sustainability loom large. How sustainable is the use of these education technologies which, after all, depend on hyperconnectivity to data? Also how sustainable are these technologies where they also depend on supply of unaffordable digital devices in these contexts marred by massive inequalities? These are problems inherent in the digitalization of education itself. These questions are important for university administrators and policy-makers to reflect on. Otherwise, digital technologies might, arguably, provide a short-term solution to the problem of access, but might in the long run, not be sustainable for fairer access amongst the lower classes whose incomes might not sustain these. The Zimbabwean case is already a clear demonstration of this.

## Conclusion

Poverty and economic instability, we conclude, breed commandist strategies in semi-authoritarian contexts whilst a relatively stable economy in a liberal democratic context breeds engagement and co-operation in times of external shocks or disruptions in the education sector. In fact, authoritarianism is symptomatic of a severe shortage of resources. The COVID-19 was thus an external shock that further disrupted an already disrupted Zimbabwean political-economic context which in turn disrupted the education sector. The COVID-19 also directly disrupted the education sector such that it found itself grappling with shocks induced by, on one hand the political-economic turmoil of post-2000 and, on the other hand the COVID-19. In as much as the university in Zimbabwe, like all the other universities elsewhere, responded by going online they could not replicate the South African universities’ example of giving staff and students both gadgets and data. They simply did not have the capacity or the willpower. Similarly, the network service providers did not provide access to any websites at zero cent charge arguably because they too have been grappling with the post-2000 economic crisis. Even though some of the MNOs provided e-learning data bundle, this remained out of reach of many of the students. Furthermore, the digital divide also impacted negatively on the students’ abilities to transit to online learning during the lockdowns in both Zimbabwe and South Africa. These were the challenges that the universities in Zimbabwe mainly faced. However, the South African case demonstrates that a relatively stable political-economic environment makes the transition to online learning relatively smooth. Finally, the findings also demonstrate that a neo-liberal political system at national level also promotes a relatively consultative management leadership style in universities whilst a relatively anti-democratic system of governance also promotes commandist management styles in universities. Thus the teaching and learning methodology shifts during the times of COVID-19 in the universities are reflective of the broader political-economic systems obtaining in Zimbabwe and South Africa and the management styles in respective universities. Overall, universities in both countries utilized a hybrid approach that used a combination of online and offline face to face methods. These methods also involve a blending of technologies and social media apps where apps such as *Zoom*, *Google* meet and *WhatsApp* all form part of the repertoire of teaching and learning tools deployed.
